# Cumulative radiation dose incurred during the management of complex pleural space infection

**DOI:** 10.1186/s12890-021-01486-7

**Published:** 2021-04-23

**Authors:** Christopher R. Gilbert, Anee S. Jackson, Candice L. Wilshire, Leah C. Horslen, Shu-Ching Chang, Adam J. Bograd, Eric Vallieres, Jed A. Gorden

**Affiliations:** 1 Thoracic Surgery and Interventional Pulmonology, Swedish Cancer Institute, 1101 Madison St, Suite 900, Seattle, WA 98104 USA; 2 Department of Surgery, Medstar Georgetown University Hospital, Washington, DC USA; 3 Medical Data Research Center, Providence St. Joseph Health, Portland, OR USA

**Keywords:** Complex pleural space infection, Cumulative effective dose, Radiation exposure, Radiation safety, Empyema management

## Abstract

**Background:**

Complex pleural space infections are commonly managed with antibiotics, pleural drainage, intrapleural fibrinolytic therapy, and surgery. These strategies often utilize radiographic imaging during management, however little data is available on cumulative radiation exposure received during inpatient management. We aimed to identify the type and quantity of radiographic studies along with the resultant radiation exposure during the management of complex pleural space infections.

**Methods:**

Retrospective review of community network healthcare system from January 2015 to July 2018. Patients were identified through billing databases as receiving intrapleural fibrinolytic therapy and/or surgical intervention. Patient demographics, clinical outcomes, and inpatient radiographic imaging was collected to calculate cumulative effective dose.

**Results:**

A total of 566 patients were identified with 7275 total radiographic studies performed and a median cumulative effective dose of 16.9 (IQR 9.9–26.3) mSv. Multivariable linear regression analysis revealed computed tomography use was associated with increased cumulative dose, whereas increased age was associated with lower cumulative dose. Over 74% of patients received more than 10 mSv, with 7.4% receiving more than 40 mSv.

**Conclusions:**

The number of radiographic studies and overall cumulative effective dose in patients hospitalized for complex pleural space infection was high with the median cumulative effective dose > 5 times normal yearly exposure. Ionizing radiation and modern radiology techniques have revolutionized medical care, but are likely not without risk. Additional study is warranted to identify the frequency and imaging type needed during complex pleural space infection management, attempting to keep ionizing radiation exposure as low as reasonably possible.

## Introduction

Parapneumonic effusions are common and are often treated with antibiotics and/or pleural drainage. Although less common, more complicated pleural disease in the setting of pneumonia, such as complicated parapneumonic effusions and empyema can carry a high morbidity and mortality and often require additional interventions. The current management of complex pleural space infections (CPSI) generally requires antibiotics, pleural space drainage, and in some cases will require additional interventions such as intrapleural fibrinolytic therapy (IPFT) and/or surgery (evacuation and/or decortication). Published guidelines exist on the management of infectious pleural disease itself [1, 2], however little attention has been paid to radiation exposure that may be incurred during the management of these processes and interventions.

Diagnostic and therapeutic imaging of the chest often includes single (portable anteroposterior) or two (posteroanterior and lateral) view chest x-ray, computed tomography of the chest, and fluoroscopy—all of which produce ionizing radiation to the patient and sometimes healthcare staff. These imaging modalities are generally considered “routine” and utilized during the initial evaluation as well as during ongoing management of pneumonia and CPSI. We are unaware of any recommendations or attempts at standardization/minimization of radiation exposure during CPSI management. Globally speaking, the medical field has moved towards minimizing radiation exposure to patients as reasonable in order to provide adequate care, as most believe there is increased risk of carcinoma with increased exposure to ionizing radiation [3]. As a result of these concerns, attention has been called to radiation during medical care in certain populations, including trauma patients [4, 5], children [6–8], ICU care [9, 10], and general hospital care [11]. However, lack of awareness and guidelines related to radiation exposure during CPSI management has the potential for significant practice variation and subsequent differences in radiographic study use and radiation exposure. We aimed to determine the number and type of radiographic studies performed and the resultant radiation cumulative effective dose during the management of CPSI within a large, multicenter healthcare network.

## Methods

### Data sources and study population

A retrospective review of patients undergoing management of complicated pleural space infection from January 2015 to July 2018 within a multistate community-based network of 18 hospitals was performed. The Swedish–Providence Health Care system is a community-based network located in the Western United States, covering Alaska, Washington, Idaho, Montana, Oregon, and California. An ethics committee, The Institutional Review Board at Swedish Medical Center approved this study, and a waiver of consent was granted (Study# 2018000200). All methods were performed in accordance with relevant guidelines, practices, and regulations related to performing human subject research at Swedish Medical Center.

All patients were initially identified from a central inpatient billing database. IPFT use was identified from pharmacy billing records of intrapleural dornase. Surgical intervention was identified from procedural codes for thoracotomy or thoracoscopy with or without decortication in the setting of chest tube insertion and diagnosis of pleural infection/empyema (Table 1).

**Table 1:** CPT and ICD billing codes associated with complex pleural space infection and related interventions

CPT code	ICD-9 code	ICD-10 code
32652	34.52	0BDN4ZZ
32651	34.51	0BDN0ZZ
32220	34.24	0BDP4ZZ
32225	34.59	0BDN0ZX
32320	510.0	0BDN3ZZ
32653	510.9	0BDN4ZX
32551	511.0	0BDP0ZZ
32556	511.1	0BDP3ZX
32557	511.9	0BDP4ZX
		0BBP0ZX
		0BBP0ZZ
		0BBN0ZX
		0BBN0ZZ
		J86.0
		J86.9
		R09.1
		J90

Crossover therapy was identified when both modalities were utilized within the same hospitalization (i.e., IPFT followed by surgery). Inclusion criteria included receiving IPFT or surgical intervention for management of a CPSI. Exclusion criteria included known malignant/paramalignant pleural effusion, hemothorax, empyema related to esophageal perforation, incomplete medical record (i.e., receiving initial care at an outside hospital network), current in situ indwelling tunneled pleural catheter, and/or prior chest surgery. Patient characteristics including demographics, management strategy for CPSI, chest tube duration, and hospital stay were collected. Chart data was also reviewed to formulate a RAPID score [12, 13] at the time of chest tube placement. Review of the electronic medical record of relevant imaging reports was performed.

### Radiation dose estimates

All radiographic studies from the initial date of CPSI identification to completion of management were captured. A brief review of relevant medical physics and the rationale for using cumulative effective dose (CED) can be identified in previous papers, including one by Kim et al. [4] identifying CED in trauma patients. As a brief synopsis, the standard value for radiation dose estimate (and often reported in medical journals) collected is the sievert (Sv) or millisievert (mSv). Other reports may describe radiation exposure units as gray (Gy), however both Sv and Gy are expressed in Joule/Kilogram and often utilized when discussing radiation and exposures—all leading to confusion, [14] as they are different. At a very basic level, the General Conference of Weights and Measures has decided that Gy be used for the *absorbed dose* (commonly utilized for radiotherapy dosing) and Sv be used for the *dose equivalent* (commonly used in radiation protection). The exposure of radiation to human tissue and its effects (cancer, etc.) depends on the magnitude of the dose equivalent/estimate [14], and hence use of Sv and mSv in many human radiation exposure papers.

References of the dose estimates for each type of imaging study was obtained from previously published references [11, 15]. Chest fluoroscopy and CT guidance for placement of chest tubes are not routinely identified with a reference radiation dose in mSV. They are time dependent procedures and the dose equivalent can vary depending on the trajectory and size of the radiation beam. Therefore, we elected to average the dose equivalent based on dose length product (DLP) and dose area product (DAP) [16] for these procedures. The CED of a patient was calculated as the sum of all effective doses.

### Statistical analysis

All data was collected and stored in Excel (Microsoft, Redmond, WA). Simple descriptive statistics were used to report demographics and outcomes. Univariate linear regression analyses were first used to identify factors associated with total CED, followed by multivariate linear regression analyses. Due to concerns related to collinearity associated with total CED and variables directly related to radiation (i.e., CT scan) we elected to remove this variable from the multivariate analysis. However did want to test the impact of image guided tube thoracostomy (CT and fluoroscopic guidance) understanding there was also likely some collinearity associated with these variables, therefore we elected to run two multivariate models. Statistical significance was defined as *p* < 0.05. All statistical analyses were performed using SPSS 24.0 statistical software package (SPSS Inc., Chicago, IL, USA) and R version 3.6.0 (R Core Team 2019).

## Results

A total of 1,640 patients were identified as meeting inclusion criteria from billing records. After chart review patients were excluded for the following reasons: malignant/paramalignant pleural effusion—201, hemothorax—186, empyema related to esophageal perforation—89, incomplete medical record—117, current in situ indwelling tunneled pleural catheter—25, and prior chest surgery—456. A total of 566 patients were therefore eligible and included in the analysis.

Patient demographics and hospital stay information is available in Table 2.

**Table 2:** Patient demographics and hospital stay information

Demographic and hospital stay information	
Age (median, IQR), years	58 (46–68)
Male/Female (n, %)	374 (66%)/192 (44%)
Community Acquired Infection (n, %)	498 (88%)
RAPID Score (median, IQR)	3 (2–4)
Body Mass Index (median, IQR)	26.5 (22.7–30.7)
Admitted to hospital with Thoracic Surgery (n, %)	479 (85%)
Hospital length of stay (median, IQR)	11 (8–16)
Intensive care unit stay (median, IQR)	1 (0–3)
Duration of chest tube drainage (median, IQR)	6 (4–9)
Duration of IPFT instillation (median, IQR)	3 (3–4)

The median age of our population was 58 (IQR 46–68) years, composed of mostly males (66%—374/566) and the majority of infections were community acquired 88% (498/566). The median RAPID score was 3 (IQR 2–4). The median overall length of stay (LOS) was 11 (IQR 8–16) days and median duration of chest tube drainage was 6 (IQR 4–9) days. The majority of patients (85%) were managed in hospitals with thoracic surgeon presence.

**Fig. 1:**
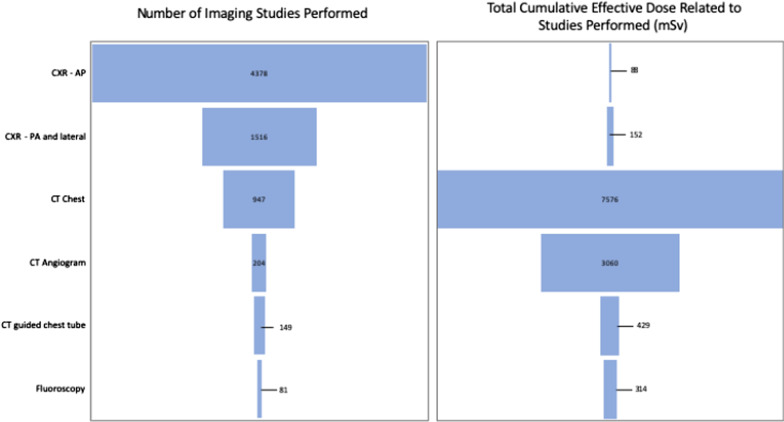
**a** Graphical representation of the total number of imaging studies performed within the population, categorized by type of imaging. The type of imaging performed is listed along the vertical axis and the total number of each imaging type is displayed within the shaded bar. **b** Graphical representation of the total cumulative dose (in millisieverts (mSv)) within the population, categorized by type of imaging. Again, the imaging modality is listed on the vertical axis and the total cumulative effective dose is listed within the shaded bar

The overall number of radiographic studies performed was 7275, with the majority of imaging, (60.2%) being a single view chest radiograph. However, the majority of radiation exposure was related to diagnostic CT imaging. Exposure to diagnostic CT imaging (combining both CT angiogram and CT chest), accounted for 92% of the total CED within our population (Fig. 1). A total of 2.5% (14/566) of the population did not receive a CT scan during their admission, whereas those undergoing CT imaging, 67.5% (382/566) underwent two or more CT scans.

**Fig. 2:**
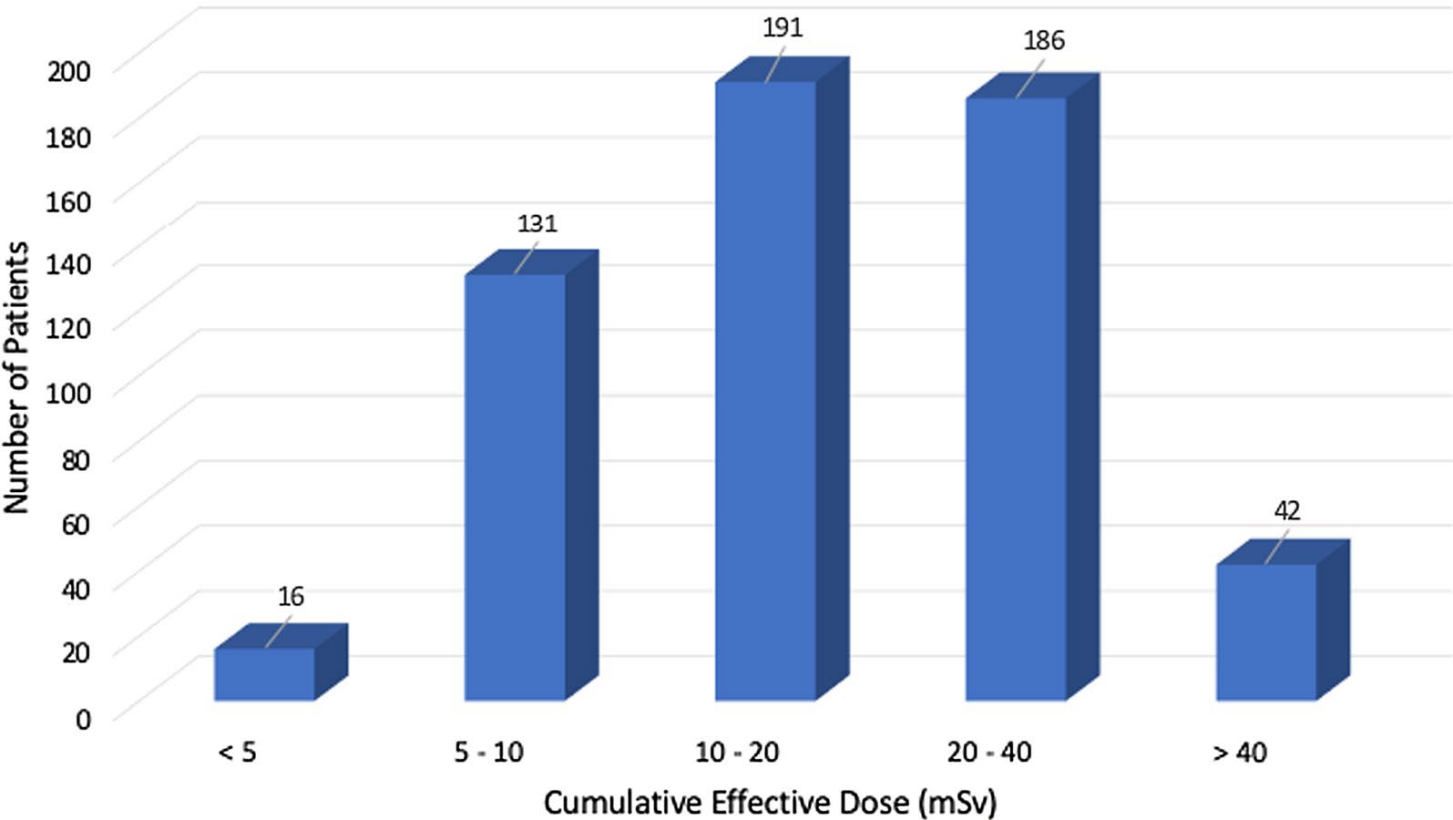
Graphical representation of the cumulative effective dose divided into exposure categories of < 5, 5–10, 10–20, 20–40, and > 40 mSv. The number of patients within each category is displayed along the vertical axis

The median CED for the entire population was 16.9 (IQR 9.9–26.3) mSv, respectively, with a range of 0.1–73.4 mSv. Over 74% of patients received more than 10 mSv during their hospital stay with 7.4% receiving more than 40 mSv (Fig. 2).

Univariate linear regression analysis (Table 3) revealed that the following variables were significantly associated with a higher CED: increased hospital length of stay (continuous variable), hospital length of stay > 7 days, total number of CT scans (continuous variable), having more than 1 CT performed, chest tube duration, evidence of abscess or necrotizing pneumonia on initial CT, and use of CT or fluoroscopic guidance for chest tube placement. The following variables were significantly associated with a lower CED; undergoing initial surgical management of CPSI, increased age, and evidence of loculation on initial CT scan. Multivariate linear regression analysis (Model 1—removal of total CT scans and more than 1 CT scan performed variables) identified that increased hospital length of stay and the use of CT or fluoroscopic guidance for chest tube placement is associated with a higher CED, whereas initial surgical management of CPSI and increased age is associated with a lower CED. When additional multivariate linear regression analysis (Model 2) is run with removal of all ionizing radiation variables (total CT scans, more than 1 CT scan performed, use of CT or fluoroscopic guidance for chest tube placement) increased hospital length of stay remains associated with a higher CED, whereas initial surgical management of CPSI remains associated with a lower CED.

**Table 3:** Univariate and Multivariate linear regression analysis

Variable	Total CED mean difference	Lower 95% CI	Upper 95% CI	p value
*Univariate linear regression analysis*				
Hospital has thoracic surgery presence (Baseline: No)	− 1.233	− 4.065	1.598	0.393
Surgical Treatment (Baseline: Medical)	− 8.446	− 10.504	− 6.388	< 0.001
Crossover Treatment (Baseline: Medical)	2.374	− 0.770	5.518	0.139
Body Mass Index	0.027	− 0.114	0.169	0.705
Body Mass Index 20–< 30 (Baseline: < 20)	1.781	− 1.999	5.561	0.355
Body Mass Index 30–< 40 (Baseline: < 20)	1.462	− 2.743	5.666	0.495
Body Mass Index > 40 (Baseline: < 20)	0.713	− 4.605	6.031	0.792
Hospital Length of Stay, Log-2 transformed, days	5.386	4.287	6.485	< 0.001
Hospital Length of Stay > 7 days (Baseline: 7 days or less)	7.404	5.060	9.749	< 0.001
Total number of CT scans	7.898	7.595	8.201	< 0.001
More than 1 CT scan (Baseline: 0 or 1 CT scan)	16.613	14.918	18.308	< 0.001
RAPID Score	0.119	− 0.546	0.785	0.725
RAPID Score = 3–4 (Baseline: 0–2)	− 1.312	− 3.507	0.882	0.241
RAPID Score = 5–7 (Baseline: 0–2)	1.072	− 1.993	4.138	0.492
Age at Admission, years	− 0.061	− 0.120	− 0.003	0.041
Chest tube duration, days	0.032	0.001	0.064	0.045
Hospital (Baseline: Community)	3.059	− 0.054	6.173	0.054
Evidence of loculation on first chest CT, yes (Ref = No)	− 2.552	− 4.928	− 0.175	0.035
Abscess or necrotizing pneumonia, yes (Ref = No)	2.935	0.173	5.696	0.037
CT guided tube thoracostomy, yes (Ref = No)	7.431	5.087	9.775	< 0.001
Fluoroscopic guided tube thoracostomy, yes (Ref = No)	8.691	5.287	12.095	< 0.001
*Multivariate linear regression analysis: model 1*				
Surgical Treatment (Baseline: Medical)	− 5.314	− 7.289	− 3.338	< 0.001
Crossover Treatment (Baseline: Medical)	2.159	− 0.714	5.032	0.140
Hospital Length of Stay, Log-2 transformed, days	3.742	2.696	4.788	< 0.001
Age at Admission, years	− 0.055	− 0.105	− 0.005	0.032
Evidence of loculation on first chest CT, yes (Ref = No)	− 1.490	− 3.527	0.548	0.152
Abscess or necrotizing pneumonia, yes (Ref = No)	− 0.326	− 2.751	2.099	0.792
CT guided tube thoracostomy, yes (Ref = No)	5.371	3.260	7.482	< 0.001
Fluoroscopic guided tube thoracostomy, yes (Ref = No)	6.388	3.334	9.442	< 0.001
*Multivariate linear regression analysis: model 2*				
Surgical Treatment (Baseline: Medical)	− 7.155	− 9.086	− 5.224	< 0.001
Crossover Treatment (Baseline: Medical)	0.974	− 1.952	3.900	0.513
Hospital Length of Stay, Log-2 transformed, days	4.162	3.094	5.230	< 0.001
Age at Admission, years	− 0.051	− 0.102	0.000	0.052
Evidence of loculation on first chest CT, yes (Ref = No)	− 1.516	− 3.615	0.583	0.156
Abscess or necrotizing pneumonia, yes (Ref = No)	0.219	− 2.273	2.711	0.863

## Discussion

We identified the number of radiographic studies and the overall CED in patients hospitalized for CPSI was high. Recent publications suggest annual environmental radiation dose exposure within the United States around 3 mSv [3, 17]. Median CED within our population was > 5 times that amount, with more than 97% of our population receiving 5 mSv or more during their admission.

Ionizing radiation utilizing modern radiology techniques has tremendous benefits, likely revolutionizing medical care in the present time. However, this improvement and access to technology is likely not without side effects or risks. There remains debate regarding the true “risk” of low level/dose radiation exposures, however the literature has some data to draw on, including data available from Japanese atomic bomb survivors. Individuals receiving dose ranges of 5–100 mSv (mean exposure of 29 mSv), had significant increases in solid-cancer incidence when compared to those exposed < 5mSv [18], suggesting some increased carcinoma risk, even with these lower dose exposures. Despite this the controversy remains as to the true “risk” associated with low dose exposure and what constitutes a low dose exposure. However, most governing bodies accept that radiation induced risks/effects are real (including doses < 100 mSv), likely have a linear relationship to exposure, and there is likely no completely “safe” dose [19]. Recommendations from both the National Council on Radiation Protection and Measurements and the International Commission on Radiation Protection suggest radiation dosing be kept as low as reasonably achievable [20], the so-named ALARA principle.

Prior studies report varying radiation exposures during hospitalizations, but most have been focused on ICU stays and/or trauma evaluations. Lutterman et al. identified 200 inpatients receiving a mean dose of 14.8 mSV, with significantly higher doses in those spending time in the ICU [11]. Moloney et al. identified 421 ICU patients receiving a median CED of 1.5 mSV, with the highest median CED found in trauma patients (7.7 mSv) [10]. Krishnan et al. identified 4155 medical ICU patients reporting a median CED of 0.72 mSV, of note, within their population, three percent did receive > 50 mSv [9]. Kim et al. identified critically ill trauma patients requiring prolong ICU stays (> 30 days), reporting a mean CED of 106 mSv per patient [4].

We similarly report on significant radiation exposure in a population of inpatients being managed for CPSI. The majority of radiation exposure (> 90% of CED) during CPSI management appears related to diagnostic CT scan imaging (CT chest or CT angiogram of the chest).

The ability to ascertain why imaging was ordered within a retrospective multi-institutional review remains a significant limitation of our study. Presumably all ordered imaging was “necessary”, however reviewing charts retrospectively does not allow for one to reliably identify the reason for ordering imaging. Reviewing progress notes as well as orders within an electronic medical record can provide documentation, however the interpretation of such information in a retrospective manner is likely unreliable and potentially misleading. Advanced imaging modalities have likely led to improved detection and the ability to successfully manage certain conditions, perhaps with CPSI being one of them. We have identified that a majority of patients being managed for CPSI are receiving large amounts of ionizing radiation, including frequent CT imaging. Prior studies, such as the MIST 2 Trial [21], identified chest x-ray imaging as a potential endpoint for determining improvement in pleural disease. Within their trial there was no dictum for utilizing chest x-ray vs CT for CPSI evaluation, rather chest x-ray was simply their primary research endpoint as an objective measure. Interventions such as modifying practice habits to limit chest imaging frequency, utilizing low dose CT imaging, or use of non-ionizing chest imaging (pleural ultrasound) may help minimize radiation exposure to patients, however the clinical impact of such a change is currently unknown. Proposals such as these would be reasonable as we would offer comparison to other situations in which radiographic imaging was considered standard, but over time have fallen to the wayside after evidence of potential harm and continued safety without imaging has been demonstrated. Examples include the use of fluoroscopy during bronchoscopy with transbronchial biopsy and daily chest x-ray use for ventilated patients in the ICU. Fluoroscopy has been and is likely still taught as the standard at many institutions, however, multiple studies have suggested it is unnecessary [22, 23]—and not using it can lead to decreased radiation exposure to both patients and staff. Daily chest x-ray use for ventilated ICU patients or after recent pulmonary resection had also been considered a “gold standard” necessary test. However, more recent data suggests that this notion may be untrue and imaging without clinical concern may be unnecessary [24, 25].

Non-ionizing radiation techniques, such as pleural ultrasound have been helpful in the management of pleural disease [26, 27], however we are unaware of any data suggesting its utility in the more longitudinal management of inpatients with CPSI and how it may fare against other modalities such as chest x-ray or chest CT. An additional limitation of our study was the inability to capture the number of ultrasound guided procedures during CPSI management. Anecdotally, we are aware that some ultrasound guided tube thoracostomy is performed, it remains unclear as to the availability and/or expertise of pleural ultrasound within a larger healthcare system network [27, 28]. It also remains unclear as to how and/or if longitudinal pleural ultrasound is utilized. Numerous variables appear associated with CED on univariate linear regression analysis. However, due to concerns related to collinearity and variables of ionizing radiation exposure we decided to remove these variables during multivariate analysis. In model 1—the use of CT and fluoroscopic guidance are associated with increased CED, however we again suggest caution in the interpretation of this related to previous concerns of collinearity. After removal of all ionizing radiation variables it appears that hospital length of stay is the most predictive of increased CED (in both models), consistent with the likely need for longer stay suggesting more complicated disease process and therefore likely additional imaging to manage more complicated disease. Initial surgical intervention and increased age remain associated with decreased CED. It remains unclear as to the association of increased age and decreased CED. We identified additional variables such as RAPID score, Age, and BMI impacted treatment modalities (data not shown) but found no significant associations. We can suggest that perhaps increased age is associated with less aggressive treatment, however as just noted, we were unable to provide any evidence to support such. In light of the retrospective nature of this study it does remain difficult to make any additional associations.

This study has several limitations. It is a retrospective review and all of the inherent bias and shortcomings associated with such studies. One shortcoming in retrospective studies is often incomplete medical records and inability to determine clinical decision making. While we were unable to perform the latter, regarding the former we feel fairly confident that the use of a system-wide, unified, electronic medical record allows us to accurately obtain our specified datapoints with tremendous accuracy. We suspect that our radiation exposure values likely vary slightly from institution and from patient, however our CED values are calculated from published values previously utilized [15] potentially making them fairly generalizable.

In conclusion, we identified a large number of radiographic studies being performed during the inpatient management of CPSI, with a resultant high CED. The median CED within our population was greater than 5 times the normal yearly radiation exposure, and 74% of the population received more than 10 mSv during their stay. While we wholeheartedly agree that modern radiology techniques have tremendous benefits and have revolutionized medical care, they are likely not without side effects or risks. We would suggest ongoing evaluation of CED during admission for CPSI, including consideration of expanding to other disease processes that may place patients and healthcare staff at excessive risk of radiation exposure. One potential alternative strategy to high radiation exposure imaging (such as standard CT scans) could include the use of widespread pleural ultrasound for both initial evaluation and longitudinal follow-up. Other potential strategies could include the use of low dose CT scan imaging for subsequent follow-up imaging, and/or protocol driven imaging attempting to limit overall radiation exposure during hospital admission. Additional study is warranted to identify the frequency and type of imaging needed in the management of complex pleural space infections as an attempt to keep ionizing radiation exposure as low as reasonably possible.

## Data Availability

The datasets analyzed during the current study can be made available by the corresponding author on reasonable request.

## Abbreviations

CPSI: Complex pleural space infections
CED: Cumulative effective dose
IPFT: Intrapleural fibrinolytic therapy
mSv: Millisievert
Sv: Sievert
Gy: Gray
IQR: Interquartile range
CT: Computed tomography
DLP: Dose length product
DAP: Dose area product
LOS: Length of stay
ALARA: As low as reasonably achievable
BMI: Body mass index
ICU: Intensive care unit
